# Lenition in L2 Spanish: The Impact of Study Abroad on Phonological Acquisition

**DOI:** 10.3390/brainsci14090946

**Published:** 2024-09-21

**Authors:** Ratree Wayland, Rachel Meyer, Sophia Vellozzi, Kevin Tang

**Affiliations:** 1 Department of Linguistics, University of Florida, Gainesville, FL 32611, USA; rmeyer2@ufl.edu; 2 Department of Computer & Information Science & Engineering, University of Florida, Gainesville, FL 32611, USA; s.vellozzi@ufl.edu; 3 Department of English Language and Linguistics, Institute of English and American Studies, Faculty of Arts and Humanities, Heinrich Heine University Düsseldorf, 40225 Düsseldorf, Germany; kevin.tang@hhu.de

**Keywords:** L2, Spanish, lenition, studying abroad, plasticity

## Abstract

**Objective:** This study investigated the degrees of lenition, or consonantal weakening, in the production of Spanish stop consonants by native English speakers during a study abroad (SA) program. Lenition is a key phonological process in Spanish, where voiced stops (/b/, /d/, /ɡ/) typically weaken to fricatives or approximants in specific phonetic environments. For L2 learners, mastering this subtle process is essential for achieving native-like pronunciation. **Methods:** To assess the learners’ progress in acquiring lenition, we employed Phonet, a deep learning model. Unlike traditional quantitative acoustic methods that focus on measuring the physical properties of speech sounds, Phonet utilizes recurrent neural networks to predict the posterior probabilities of phonological features, particularly sonorant and continuant characteristics, which are central to the lenition process. **Results:** The results indicated that while learners showed progress in producing the fricative-like variants of lenition during the SA program and understood how to produce lenition in appropriate contexts, the retention of these phonological gains was not sustained after their return. Additionally, unlike native speakers, the learners never fully achieved the approximant-like realization of lenition. **Conclusions:** These findings underscore the need for sustained exposure and practice beyond the SA experience to ensure the long-term retention of L2 phonological patterns. While SA programs offer valuable opportunities for enhancing L2 pronunciation, they should be supplemented with ongoing support to consolidate and extend the gains achieved during the immersive experience.

## 1. Introduction

Mastering the sounds of a second language (L2) as an adult is often daunting, primarily due to the interference of the phonetic and phonological rules ingrained in one’s native language (L1). Adult learners frequently encounter difficulties perceiving and producing sounds that are absent or treated differently in their first language, leading to persistent foreign accents and miscommunications. One particularly challenging aspect for English speakers learning Spanish, for instance, is the phenomenon of lenition, where certain consonants are weakened in specific phonetic environments. This subtle process is difficult to perceive and reproduce accurately, often resulting in foreign-accented pronunciation that can hinder effective communication.

Despite these challenges, the potential for improvement exists. Evidence from both naturalistic and structured learning environments, such as study-abroad programs and laboratory training, indicates that adult learners can significantly enhance their L2 speech production. These improvements are not merely superficial, but reflect deeper changes in the brain’s processing of speech sounds, suggesting that the adult brain remains plastic and capable of adapting to new linguistic demands. The success of such interventions underscores the brain’s remarkable ability to reorganize itself in response to intensive language exposure and training, offering hope that even late learners can achieve proficiency in L2 phonetics with the right support and methodologies.

Lenition, where voiced stops /b, d, ɡ/ typically transform into approximants [$\underset{˕}{\beta }$, $\underset{˕}{ð}$, $\underset{˕}{ɣ}$] in intervocalic positions, is a common and significant feature of Spanish phonology. This process presents a challenge for second language (L2) learners who need to master these subtle articulatory changes to achieve native-like pronunciation. This study explored whether immersive experiences in a Spanish-speaking environment, such as a study abroad-program, can enhance L2 learners’ ability to produce these lenited forms. Understanding lenition, which operates as a gradient process influenced by factors such as syllable stress and surrounding vowel environment, is crucial for L2 learners aiming to refine their phonological skills in Spanish.

## 2. Neuroplasticity and Language Learning

Neuroplasticity, the brain’s ability to reorganize itself by forming new neural connections, is fundamental to both learning and recovery from brain injury. The authors of [1] emphasized that this experience-dependent plasticity plays a critical role in acquiring new skills and rehabilitation after brain damage. The influence of neuroplasticity on cognitive skills is well illustrated in a classic study [2], which found that licensed London taxi drivers exhibited significant structural differences in their hippocampi compared to control subjects. Specifically, taxi drivers had a larger posterior hippocampus, correlating with their extensive navigational experience, while the control group showed greater anterior hippocampal volume. These structural differences suggest localized plasticity in the adult brain driven by environmental exposure. This study laid the groundwork for understanding how extensive, experience-based learning can lead to structural changes in the brain, a concept further explored in language learning contexts.

Expanding on the idea that experience can induce structural brain changes, research on second language (L2) acquisition has shown that neural adaptations occur in response to new linguistic demands. For instance, Ref. [3] demonstrated how intensive training in distinguishing the English /r–l/ contrast led to significant improvements in identification performance among adult native Japanese speakers.

Specifically, it investigated how adult native Japanese speakers, who typically struggle to distinguish the English /r–l/ contrast, exhibited changes in brain activity after undergoing extensive perceptual training. fMRI was used to examine brain activity before and after a month-long training program designed to improve the identification of the /r–l/ contrast. The results showed that training led to significant improvements in identification performance, which were accompanied by enhanced brain activity not only in regions associated with acoustic-phonetic processing, such as the superior and medial temporal areas, but also in additional cortical and subcortical regions involved in auditory-articulatory mappings, including the supramarginal gyrus, planum temporale, Broca’s area, and the cerebellum. Surprisingly, brain activity for a difficult contrast did not become more similar to that of an easy contrast. Instead, the perception of a difficult L2 phonetic contrast was facilitated by the recruitment of neural processes involved in speech motor control and auditory–articulatory integration. This suggested that perceptual learning of challenging phonetic contrasts involves a complex reorganization and expansion of neural resources, reflecting the brain’s adaptive capacity to improve speech perception through experience and training. Ref. [4] provided further evidence for the reallocation of neural substrates as a result of phonetic learning. Their fMRI study demonstrated that after training with a nonnative Hindi dental-retroflex contrast, monolingual English speakers recruited brain regions typically associated with processing native contrasts. These findings suggested that successful phonetic learning in adults can involve the recruitment and efficient use of neural substrates originally dedicated to native language processing and that the adult brain can optimize its neural resources for new linguistic challenges.

Ref. [5] explored how the timing of L2 acquisition affects brain structure, specifically cortical thickness, in bilingual and monolingual individuals. The study involved 88 participants, including 22 monolinguals and 66 bilinguals, who were categorized into three groups based on when they learned their second language: simultaneous bilinguals (ages 0–3), early sequential bilinguals (ages 4–7), and late sequential bilinguals (ages 8–13). The researchers found that later L2 acquisition was associated with increased cortical thickness in the left inferior frontal gyrus (IFG) and decreased thickness in the right IFG. Early and late sequential bilinguals exhibited these structural changes, whereas simultaneous bilinguals showed no significant differences compared to monolinguals, indicating that acquiring two languages from birth does not alter brain structure in the same way as learning a second language later in childhood. The study also revealed that the age of L2 acquisition significantly correlated with cortical thickness within the bilingual group: the later the L2 was acquired, the thicker the cortex in the left IFG and the thinner it was in the right IFG. These findings suggested that learning a second language after proficiency in a first language induces specific structural changes in the brain, particularly in the IFG, and that the age of acquisition plays a crucial role in shaping these changes.

More recently, Ref. [6] examined whether extensive L2 experience can lead to neuroplastic changes that improve the ability to distinguish the English /ɪ/ and /i:/ contrast, as in “ship” versus “sheep” among native French speakers. The experiment was conducted with 28 intermediate English-speaking French adults, focusing on how their ability to discriminate L2 phonemic contrasts influenced the processing of semantic violations in sentences. The results revealed three ERP effects related to L2 proficiency: a left frontal N100, a reduced phonological mismatch negativity (PMN), and a semantic N400. These findings suggested that neuroplasticity enables late acquisition of difficult linguistic features, such as phonemic contrasts, and highlights the interaction between phonological and semantic processing during language comprehension.

Ref. [7] extended these insights by exploring how adults can learn to integrate pitch patterns into words, a critical ability for mastering tonal languages like Mandarin among native speakers of English. Their findings revealed that successful learners showed increased activation in language processing areas, while less successful learners activated regions typically involved in nonlinguistic pitch processing. This study highlights how individual differences in neural adaptation can influence language learning success, offering a nuanced view of how neuroplasticity manifests differently across learners.

Following Ref. [7]’s exploration of pitch pattern learning, Ref. [8] investigated oscillatory brain networks after short-term lexical tone training. Their EEG study demonstrated that native English speakers, unfamiliar with tonal languages, developed new neural networks to accommodate tonal distinctions. This finding further underscores the brain’s capacity to reorganize in response to new linguistic demands, particularly when exposed to unfamiliar phonetic contrasts.

Ref. [9] provided a comprehensive review of the mechanisms and timeline of experience-driven changes in the neural circuits involved in speech acquisition and learning. The authors discussed the critical period hypothesis, which posits that there is an optimal window for language learning during early childhood when the brain’s plasticity is most pronounced. However, they argued that neural plasticity continues into adulthood, allowing for the acquisition of new languages, though with more difficulty compared to early childhood. They emphasized the use of modern brain imaging techniques to explore brain–behavior connections in language learning, focusing on measures such as neural sensitivity, efficiency, specificity, and connectivity.

The review also examined how early exposure to a first language shapes neural circuits in ways that can either facilitate or hinder second-language acquisition, introducing the concept of native language neural commitment. This neural commitment enhances processing efficiency for native language sounds, but reduces sensitivity to nonnative phonetic contrasts. The authors presented evidence from phonetic training studies, particularly focusing on training Japanese speakers to distinguish between the English /r/ and /l/ sounds, phonemes that are difficult for Japanese learners due to the lack of similar distinctions in their native language. The training led to significant improvements in learners’ ability to identify and discriminate the /r/ and /l/ sounds, with a 20% increase in identification accuracy. Neuroimaging data revealed that this improvement was accompanied by increased neural sensitivity to the /r/–/l/ distinction, particularly in the left hemisphere, and enhanced neural efficiency, as evidenced by reduced activation spread and shorter activation duration. These findings underscore the potential for significant neural plasticity in adulthood, suggesting that with enriched linguistic experience and targeted training, adults can achieve meaningful improvements in phonetic category acquisition.

Similarly, Ref. [10] provided a comprehensive review of L2-induced neuroplastic changes, showing that bilingual experiences can lead to increased gray-matter density and improved white-matter (WM) integrity across various age groups. Their findings suggest that even short-term language training can induce significant neural changes, challenging the notion that plasticity is limited in adulthood. The implications of this research underscore the rapid adaptability of the brain, building upon the foundational work of previous studies and reinforcing the idea that the adult brain remains highly plastic.

The review highlighted that these structural changes can be observed across different age groups, from children to the elderly, and can occur rapidly, even with short-term language training. For instance, studies have shown that increased gray-matter (GM) density in the left inferior parietal lobule (IPL) is associated with bilingualism, with greater changes seen in individuals who acquire their second language earlier in life. Similarly, short-term L2 training, such as intensive vocabulary learning over 16 weeks, has been shown to increase GM volume in the right IFG and enhance WM connectivity between the IFG and other brain regions like the caudate nucleus and superior temporal gyrus.

The authors also addressed individual differences in brain structure that may influence L2 learning success. For example, variability in the left Heschl’s gyrus (HG) has been linked to differences in phonological learning abilities, with greater GM volume in this region correlating with better performance in tasks like learning lexical tones. The authors suggested that these individual differences, combined with factors like the intensity and quality of language exposure, play a crucial role in shaping the brain’s response to L2 learning.

Together, these studies and reviews demonstrated the extensive scope of neuroplasticity, from its role in general cognitive functions to the specific challenges of language learning and pronunciation. They underscored the brain’s remarkable ability to adapt and reorganize in response to new linguistic experiences, offering valuable insights into the mechanisms underlying successful language acquisition and the potential for targeted, effective language training strategies.

## 3. Lenition in L2 Spanish Learners and Effects of Studying Abroad

Lenition, or consonant weakening, is a prevalent phonological feature in Spanish. In most non-word-initial environments, voiced stops /b, d, ɡ/ are lenited to fricatives [β, ð, ɣ] or approximants [$\underset{˕}{\beta }$, $\underset{˕}{ð}$, $\underset{˕}{ɣ}$] [11]. This lenition process is gradient-based, meaning that stops can surface with varying degrees of lenition, ranging from fricatives to approximants or even being deleted entirely. This lenition process is gradient-based, meaning that stops can surface with varying degrees of lenition, ranging from fricatives to approximants or even being deleted entirely [12].

For L2 learners, mastering the production of these voiced stop allophones can be challenging [13]. These difficulties are compounded by the influence of their native language phonology, the extent of their exposure to Spanish, and their overall proficiency. For example, Ref. [14] found that English speakers struggle with the lenition of Spanish voiced stops /b, d, ɡ/ due to the lack of an allophonic lenition rule in English, slower acquisition of the phone [ð] compared to [β] and [ɣ], and confusion caused by the orthographic similarity of <b> and <v>. These challenges were more pronounced in formal reading tasks than in casual conversation. Ref. [15] observed that the pronunciation of stops and approximants in Spanish by English speakers varied according to their Spanish proficiency, with more advanced learners adopting native-like phonetic cues. Their findings suggested that as learners gain experience, they begin to approximate native speaker patterns more closely. Expanding on this idea, Ref. [16] applied an optimality-theoretic framework to examine how English speakers acquired voiced stop lenition in stages that correspond to the prosodic hierarchy. Her study demonstrated that as proficiency increases, learners tend to lenite stops to approximants more readily at syllable and prosodic word onsets, showing a progression toward native-like behavior.

Further exploring the variability in learner outcomes, Ref. [17] tracked the progression of Spanish [β] pronunciation among English-speaking learners over a year. His study revealed diverse trajectories, with some learners showing improvements and others experiencing regressions. This variability highlights the dynamic nature of L2 phonological development. Similarly, Ref. [18] compared the lenition of Spanish voiced stops between low-intermediate and advanced English-speaking learners and native speakers. Her findings indicated that advanced learners exhibited more lenition, particularly in word-internal and non-stressed positions. The author also noted the impact of orthography on phoneme production, with notable differences in the handling of <b> and <v>, further complicating the acquisition process for L2 learners. Collectively, these studies underscore the complexity of lenition for L2 learners, showing that both phonetic and orthographic factors significantly influence their acquisition of Spanish phonology.

The impact of study abroad (SA) programs on L2 phonological acquisition, particularly lenition, has also been explored, though with mixed results. A systematic review [19] examined the effectiveness of SA programs on linguistic proficiency gains among undergraduate language learners, highlighting that while SA programs are particularly effective in improving oral fluency and general linguistic proficiency, evidence for gains in other areas like written accuracy, reading, and grammar is limited. This review underscored challenges such as small samples, lack of randomization, and variability in measures, which complicate efforts to draw strong conclusions about the impact of SA on language development. Despite these limitations, the findings suggested that SA experiences provide valuable opportunities for learners to enhance their oral fluency and speaking confidence, largely due to increased opportunities for interaction in immersive environments.

Additionally, Ref. [20] provided a comprehensive review of research on the impact of SA on L2 phonetic and phonological development. They highlighted methodological challenges, such as the balance between control and naturalness in data elicitation tasks, and revealed that while some learners improve their pronunciation during SA, these gains are not universal. The acquisition of region- and dialect-specific features is inconsistent and individual variation is significant, emphasizing the need for further research to understand how factors like L1–L2 pairings and SA duration influence phonological development.

Specific studies focused on lenition have shown that SA can facilitate the acquisition of phonetic features less likely to be emphasized in classroom settings. One notable study [21] investigated the combined effects of immersion and explicit instruction on acquiring Spanish phonology by native English speakers, focusing on fricative ([β], [ð], [ɣ]) and occlusive ([b], [d], [ɡ]) allophones of /b/, /d/, and /ɡ/. In this study, participants were divided into two groups: those who had received prior phonetic instruction and those who had not, with both groups participating in an eight-week immersion program in Mexico. The findings revealed that while both groups performed well on occlusive sounds due to positive transfer from English, significant differences emerged in the production of fricative allophones. The instruction group showed higher pre-immersion accuracy (8.6%) compared to the no-instruction group (3.3%), and both groups improved post-immersion, with the instruction group achieving 28.7% accuracy and the no-instruction group 5.8%. Despite these gains, neither group reached native-like proficiency, particularly with fricatives. The study concluded that the combination of instruction and immersion is beneficial, but insufficient for achieving high phonological accuracy, highlighting the need for further research and suggesting that language programs should integrate both strategies to optimize pronunciation outcomes.

Similarly, Ref. [22] found that students who studied abroad showed greater use of lenited variants of /s/ in Spanish, indicating that the SA context provides crucial opportunities for learners to acquire phonetic features that are less likely to be emphasized in formal classroom settings. This underscored the potential of SA programs to enhance phonological development, though the gains may be modest and vary depending on the specific linguistic features being acquired.

In conclusion, this body of research indicated that lenition is a complex aspect of Spanish phonology that presents significant challenges for L2 learners. While study-abroad programs offer a valuable context for acquiring these features, particularly through immersion, the results are often variable and depend on numerous factors, including prior instruction, the length of immersion, and individual learner differences. The combination of explicit phonetic instruction and immersion appears to be beneficial, though not sufficient on its own to achieve native-like pronunciation. Future research should continue to explore these dynamics with larger, more diverse samples and consider longitudinal designs to better understand how learners can be supported in acquiring challenging phonological features like lenition.

## 4. This Study

Lenition impacts acoustic properties such as intensity, duration, and periodicity, with intensity serving as a key measure. Research has shown that smaller intensity differences between consonants and adjacent vowels indicate more pronounced lenition. Additionally, metrics like maximum rising velocity, mean intensity, and relative duration of consonants are used to evaluate lenition, revealing that more lenited sounds typically have shorter durations and distinct intensity profiles. The harmonics-to-noise ratio (HNR) also serves as an indicator, with a higher HNR suggesting a vowel-like quality in more lenited sounds.

In a departure from previous methods, this study introduces a novel approach to quantify the lenition of the Spanish voiced stop consonants /b, d, ɡ/ among native speakers of English throughout a study-abroad program using a deep learning model, Phonet, that calculates the posterior probabilities of phonological features relevant to lenition, specifically continuant and sonorant, from acoustic data [23].

### 4.1. Phonological Features and Lenition

Phonemes are categorized based on shared phonetic traits. The broadest classification is [consonantal], where phonemes in most languages are either [+consonantal], involving some constriction of the vocal tract, or [-consonantal], without such constriction. The [+consonantal] group includes stops, fricatives, affricates, nasals, and liquids, while vowels and glides are typically [-consonantal]. The [syllabic] class contrasts with [consonantal]: [+syllabic] phonemes, such as vowels and certain consonants (e.g., 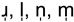), form the nuclei of syllables, unlike [-syllabic] consonants and glides.

The next major class, [sonorant], includes phonemes like nasals, liquids, glides, and vowels that allow relatively free airflow and resonance. This contrasts with [+obstruent] sounds, such as stops and fricatives, which are produced with a substantial or complete obstruction of airflow through the vocal tract. The [continuant] class involves sustained airflow: [+continuant] sounds, including fricatives, liquids, glides, and vowels, allow ongoing airflow despite partial closure. Nasals, while often classified as [-continuant] due to the blockage of oral airflow, are considered [+continuant] because they allow nasal airflow (see [24] for further details on phonological features).

Phonological features categorize phonemes into natural classes that share specific traits, influencing their behavior in phonological processes. For instance, in English, /p, t, k/ share multiple negative features, such as [-syllabic, -voice, -continuant, -sonorant, -delayed release], and they aspirate in certain positions. Similarly, Spanish /b, d, ɡ/ are grouped together, but undergo lenition, changing their features to become more fricative-like [+continuant] and/or approximant-like [+sonorant] in specific contexts. However, to fully capture the varied and gradual degrees of lenition, it is essential to go beyond the categorical manifestations of these changes.

### 4.2. Phonetic Gradience and Posterior Probability

Computational methods have been employed to capture gradient phonetic variation, such as pronunciation changes in code-mixed Hindi English speech (e.g., [dʒ]–[z] and [p^h^]–[f] variations) [25], “g”-dropping in English [26,27], and other phenomena like “th”-fronting, “td”-deletion, and “h”-dropping in English [28]. These studies often utilize forced alignment systems, which take word-level transcriptions and reference pronunciation dictionaries to suggest probable pronunciations based on acoustic properties. For instance, to capture “th”-fronting, words susceptible to this variation might be assigned two pronunciations ([θ] and [f]) in the dictionary. Based on the acoustic evidence, a trained forced aligner selects the most probable pronunciation.

Ref. [29] proposed a novel method to capture more nuanced variations, such as the degree of /l/-darkness in American English. Instead of relying on phone labels, they used log probability scores from forced alignments to measure these variations. This approach was extended to examine finer variations of /l/ in [30]. Their results highlighted both categorical distinctions and finer degrees of /l/-darkness depending on the phonetic contexts.

Other approaches, such as support vector machines (SVMs), have been used to classify r-full and r-less tokens in English based on mel-frequency cepstral coefficients (MFCCs) as acoustic representations [31]. A similar approach using random forest classification has also been applied to code sociophonetic variables in English [32]. These models, trained on canonical pronunciations, estimate variable realizations based on acoustic similarities.

The study by [33] demonstrated the potential of different modeling methods to capture lenition processes, suggesting that comparing surface forms can effectively represent various lenition processes, irrespective of their underlying forms. Various processes recognized as lenition were modeled using a spoken corpus of American English. Three distinct modeling methods were explored, each differing in how it represented the surface segments. The first method compared the surface forms of two segment types (e.g., [t] and [d] for the lenition process /t/→[d]) without considering their underlying forms. The second method focused only on surface forms that shared the same underlying form (e.g., both [t] and [d] segments originating from /t/). The third method evaluated only unchanged segments, such as [t] tokens produced from /t/ and [d] tokens from /d/. Remarkably, all three approaches produced consistent results, indicating that various acoustic manifestations of a lenition process, such as /t/→[d], can be effectively captured by comparing relevant pairs of surface segments, irrespective of their underlying forms.

The Phonet approach broadens the study of lenition beyond individual segment comparisons to encompass entire classes of segments defined by specific phonological features. Unlike the method used by [33], which focuses on segment pairs, this approach categorizes segments into groups based on binary phonological features. Specifically, it assesses the probability of the [continuant] feature, which distinguishes stops from non-stops (e.g., stops becoming fricatives), and the [sonorant] feature, which separates stops and fricatives from non-stops and non-fricatives (e.g., stops becoming approximants). These features encapsulate the primary categorical outcomes of stop lenition in Spanish. A high [continuant] probability coupled with a low [sonorant] probability indicates a fricative-like transformation, while both high [continuant] and [sonorant] probabilities suggest an approximant-like realization. This approach diverges from the method of [29,30], which calculated phonetic variation from log probability differences between two alignments (dark /l/ vs. light /l/). Instead, it reflects degrees of lenition through the probabilities of phonological features derived from the acoustic properties of the signals.

### 4.3. Phonet

Phonet’s posterior probabilities enable a gradient analysis of phonological features, complementing traditional acoustic measures [12]. Phonet has also been shown to be effective at measuring the degree of lenition [34,35]. Inputs to Phonet consist of feature sequences derived from log-energy distributed across triangular mel filters, calculated from 25 ms windowed frames of 0.5 s chunks of the input signal. These sequences are processed by two bidirectional GRU layers, which model both past (backward) and future (forward) states of the sequence simultaneously. The sequences from the second bidirectional GRU layer are then processed through a time-distributed, fully connected dense layer, resulting in an output sequence of the same length as the input. The final classification is made by a time-distributed output layer with a softmax activation function, which assigns a phonological class to each sequence. Phonet has proven highly effective in detecting phonemes and phonological classes in Spanish and in modeling speech impairments in Parkinson’s disease patients [23].

In our study, 23 phonological classes of Spanish were trained using a network of 23 Phonet systems, and 26 phonemes were trained using a single network, all optimized with an Adam optimizer [36]. Following [23], a weighted categorical cross-entropy loss function was employed to counteract class imbalance during training. For further details on the model structure and training, please refer to [12].

### 4.4. Data

The data came from the LANGSNAP project [37,38]. Twenty-seven native English speakers enrolled in a language program at a British university participated in the project. In their third year (of four), these students, who were learning Spanish, studied abroad in either Spain (n = 18) or Mexico (n = 9). Additionally, ten native Spanish speakers from Spain (n = 8) and Mexico (n = 2) who were studying in England provided comparison data.

All native English-speaking participants were in the third year of a Spanish degree program at the time of the pre-test and completed a delayed post-test one year after returning from their placements. The average age of onset for learning Spanish was 15.14 years (SD = 3.44), with ages ranging from 11 to 22. Additionally, 21 participants had studied French before university, 7 had studied German, and 4 had studied other languages, with several continuing these languages at university.

Participants experienced three types of placements abroad: studying at a university (n = 9, all in Spain), serving as English teaching assistants (n = 16, with 7 in Spain and 9 in Mexico), and workplace internships (n = 2, both in Spain). All participants completed three picture description tasks. They were shown a series of pictures and asked to narrate the story depicted in their own words. The native Spanish-speaking participants completed the task once for each of the three sets of pictures. In contrast, the study-abroad participants completed each task for each set of pictures twice over the course of the project, for a total of six time points: pretest, three times while abroad (approximately every three months), posttest, and a delayed posttest (one year after the program).

The number of tokens for each target phoneme in word-initial and word-medial positions is displayed in Table 1.

### 4.5. Methods

Audio and transcripts from the LANGSNAP project were force-aligned using the Spanish Montreal Forced Aligner [39]. The force-aligned audio and text grids served as the input for the Phonet model. The model demonstrated high accuracy in predicting continuant (91%) and sonorant (92%) features. These posterior probabilities for each feature were generated in 10 ms frames. For data analysis, phones longer than two frames were filtered to include only the middle third of the phone. The posterior probabilities for these frames were then averaged. To ensure an environment conducive to lenition, only intervocalic stops were included.

### 4.6. Statistical Analysis

Linear mixed-effect regression models [40] were conducted in R [41] with continuant and sonorant posterior probabilities as the dependent variables. The predictor variables included session (the six study-abroad time points, plus native speakers), syllable stress, stop voicing, stop place of articulation, word position (word-initial, word-medial), preceding vowel height (high, mid, low), and following vowel height. Session, place of articulation, and vowel heights were forward-difference-coded, while the other variables were contrast-coded.

## 5. Results

### 5.1. Continuant Posterior Probability

**Figure 1:**
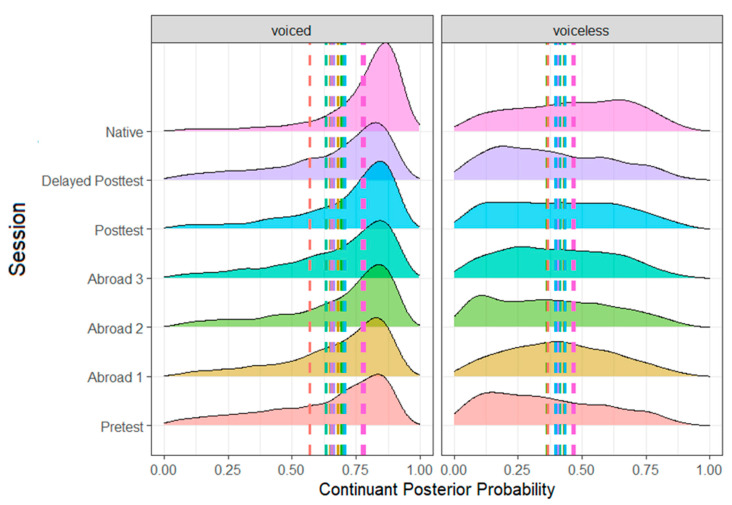
Continuant posterior probability of voiced and voiceless stops across all groups.

**Figure 2:**
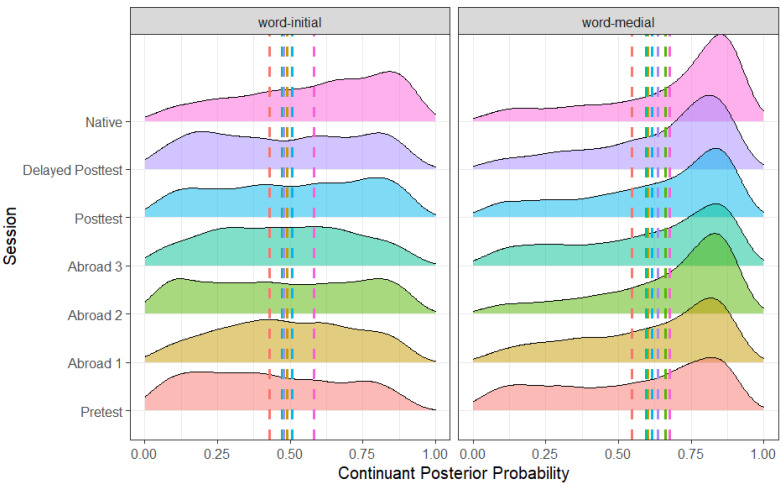
Continuant posterior probability of word-medial and word-initial stops across all groups.

As expected, there were effects of voicing (Figure 1), stress, and word position (Figure 2). Voiced stops had a higher continuant posterior probability than voiceless stops (*b* = −0.262, SE = 0.005, t = −48.477, *p* < 0.001), word-medial stops likewise had a higher continuant posterior probability than word-initial stops (*b* = 0.078, SE = 0.006, t = 14.138, *p* < 0.001), and stops in unstressed syllables had a higher continuant posterior probability than stops in stressed syllables (*b* = −0.016, SE = 0.005, t = −3.053, *p* = 0.002). There was also an effect of place, with dental stops having a higher continuant posterior probability than both bilabial (*b* = −0.013, SE = 0.006, t = −2.240, *p* = 0.025) and velar stops (*b* = 0.030, SE = 0.007, t = −4.322, *p* < 0.001). Finally, there was also an effect of the vowel adjacent to the target stop. Stops followed by mid-vowels had a higher continuant posterior probability than stops followed by open vowels (*b* = −0.262, SE = 0.005, t = −48.477, *p* < 0.001). Similarly, stops preceded by mid-vowels had a higher continuant posterior probability than stops preceded by close vowels (*b* = −0.011, SE = 0.005, t = −2.112, *p* = 0.035).

**Figure 3:**
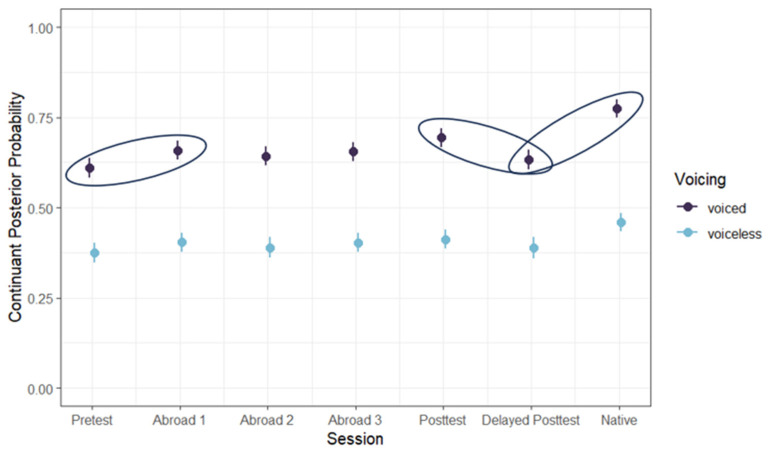
Continuant posterior probability across groups and voicing conditions. Significant comparisons are circled.

Between the pre-test and the first test abroad, there was a significant increase in posterior probability (pre-test minus first test abroad) (*b* = −0.039, SE = 0.017, t = −2.221, *p* = 0.026), showing some evidence of acquisition of lenition at least to a fricative-like production. However, there was a significant decrease in posterior probability between the post-test and delayed post-test (post-test minus delayed post-test) (*b* = 0.042, SE = 0.018, t = −2.387, *p* = 0.017), suggesting that participants did not retain the progress they made during their study-abroad program. Finally, there was a significant difference between the delayed post-test and the native speaker group (*b* = −0.107, SE = 0.017, t = −6.230, *p* < 0.001), showing that continuant posterior probability never rose to native-like levels (Figure 3).

### 5.2. Sonorant Posterior Probability

**Figure 4:**
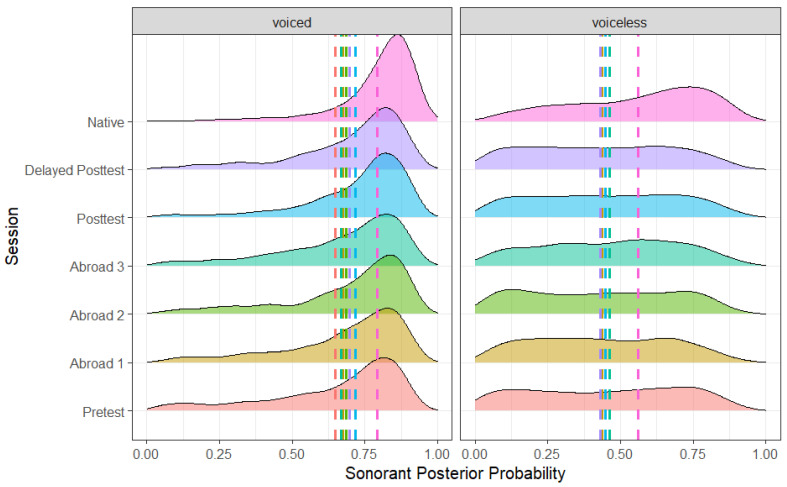
Sonorant posterior probability of voiced and voiceless stops across all groups.

**Figure 5:**
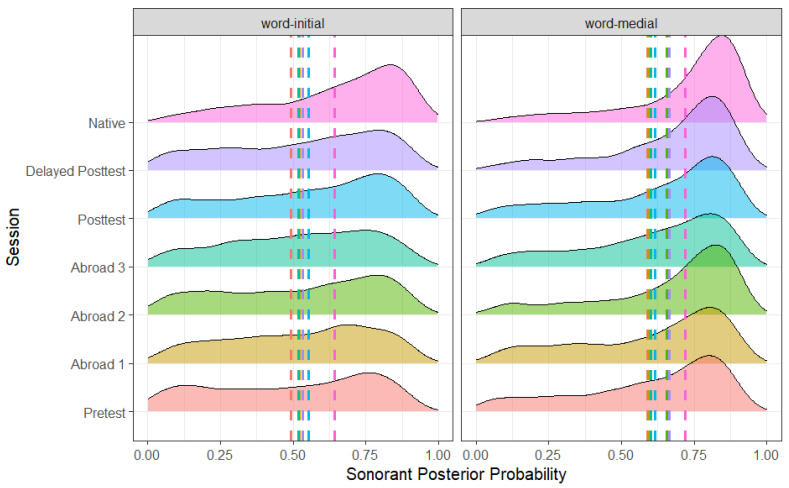
Sonorant posterior probability of word-medial and word-initial stops across all groups.

**Figure 6:**
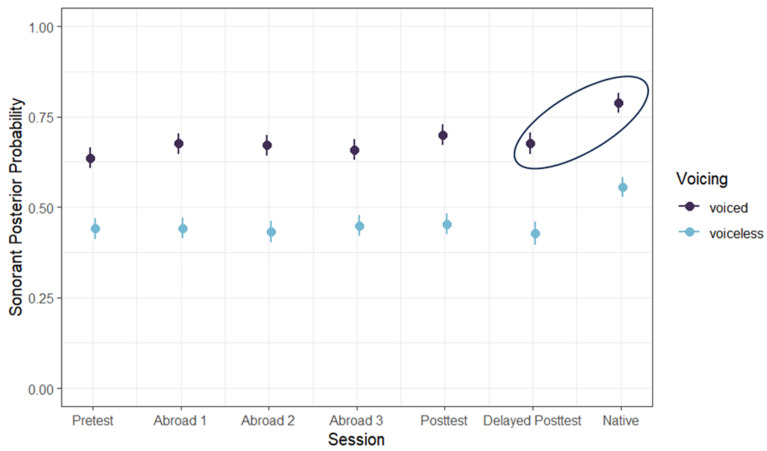
Sonorant posterior probability across groups and voicing conditions. Significant comparisons are circled.

As expected, there was an effect of word position (Figure 4) and stop voicing (Figure 5), with word-medial stops having a higher posterior probability than word-initial stops (*b* = 0.047, SE = 0.005, t = 8.784, *p* < 0.001) and voiced stops having a higher posterior probability than voiceless ones (*b* = −0.229, SE = 0.005, t = −43.655, *p* < 0.001). Unlike the results for continuant posterior probability, there was no effect of stress. In terms of place, there was a significant difference between bilabial and dental stops, with bilabial stops having a higher sonorant posterior probability (*b* = 0.014, SE = 0.005, t = 2.567, *p* = 0.010). The preceding vowel also affected the sonorance of the stop: when preceded by a mid-vowel, stops had a higher posterior probability than when preceded by a close vowel (*b* = −0.026, SE = 0.005, t = −5.066, *p* < 0.001). There was no difference between any of the L2 groups (Figure 6). However, there was a significant difference between the delayed post-test and native groups (*b* = −0.121, SE = 0.019, t = −6.419, *p* < 0.001), suggesting that L2 speakers never lenited to the level of approximants.

## 6. Discussion and Conclusions

This study explored the effects of a study-abroad program on the acquisition of lenition in Spanish voiced stops among native English speakers. The findings revealed that while learners exhibited some progress in adopting lenition patterns during their time abroad, the degree of mastery achieved was limited and the retention of these phonological patterns following study abroad was not sustained. These results highlight the complex nature of L2 phonological acquisition and raise important considerations about the design and effectiveness of study-abroad programs as a means to enhance linguistic proficiency.

One of the key findings was the significant increase in continuant posterior probability during the study-abroad period, suggesting that learners became more adept at producing fricative-like lenited forms of Spanish voiced stops. This improvement indicated that immersive exposure to a native Spanish-speaking environment can facilitate the learning of subtle phonological processes, which might be difficult to acquire in a classroom setting. The naturalistic and continuous exposure to native speech, combined with the necessity to communicate effectively in the target language, likely drove these improvements. The finding aligned with previous research showing that study-abroad programs can lead to notable gains in L2 phonetic and phonological skills, particularly in areas requiring fine-grained articulatory adjustments (e.g., [21,22]).

However, the decline in continuant posterior probability following the return from abroad raised questions about the long-term effectiveness of such programs. The fact that learners reverted to more stop-like productions after leaving the immersive environment suggested that the gains made during the program were not robustly internalized. This could be due to several factors, including the lack of continued exposure to the target language, reduced opportunities for practice, and the reactivation of L1 phonological rules upon returning to an English-dominant environment. This finding underscored the importance of sustained practice and exposure to maintain and consolidate the phonological gains made during study abroad.

These findings can be further understood in the context of neuroplasticity research associated with language learning. The increase in continuant posterior probability during the study-abroad period can be viewed as a manifestation of neuroplasticity, where learners’ brains adapted to the new phonological demands of the Spanish language. This adaptation likely involved structural changes in brain regions critical for language processing, such as the inferior frontal gyrus (IFG) and superior parietal lobule (SPL), as highlighted by [5]. However, the subsequent decline in these gains following study abroad suggests that these neuroplastic changes may not have been robust or permanent, particularly when learners returned to environments dominated by their first language (L1).

In [5], a discussion on the role of white-matter integrity in managing dual-language systems offers a possible explanation for the variability in lenition retention observed among participants. Learners’ ability to switch between L1 and L2 phonological patterns may depend on the efficiency of their white-matter tracts, particularly those connecting language-related regions. If these tracts were less developed or less efficient, this could contribute to the decline in lenition retention after the study-abroad experience, as the cognitive control mechanisms required to sustain these gains may not have been fully established.

The attention model of [42] provides additional insights, emphasizing the importance of linguistic awareness in successful L2 phonological learning. The initial gains observed during the study-abroad program suggest that learners were beginning to develop this awareness. However, the subsequent decline in performance indicates that this awareness may not have been sufficiently reinforced or that learners reverted to L1 phonological patterns once they were no longer immersed in the L2 environment. This underscores the need for targeted post-study-abroad interventions that focus on reinforcing L2 phonological patterns and enhancing learners’ ability to maintain these patterns over time.

Additionally, the role of social interaction and motivation in language learning among older adults, as discussed by [43], may also be relevant in the context of study-abroad programs. While the immersive nature of these programs provides ample opportunities for social interaction in the L2, the decline in lenition following study abroad suggests that these interactions may not have been sufficient to fully internalize the phonological patterns. This highlights the importance of continued social interaction and practice in the L2 after returning to the L1 environment, which could be facilitated through online platforms, language exchange programs, or other structured forms of practice.

The results regarding the effects of place of articulation on lenition provided further nuance. Consistent with [44]’s finding among L2 Spanish learners in Los Angeles, this study found that dental stops exhibited higher continuant posterior probabilities than bilabial or velar stops, indicating that dental stops were more likely to undergo lenition. This finding suggested that the L1 transfer facilitates the acquisition of this sound in L2 Spanish in a classroom, as well as in a study-abroad setting.

In contrast, the results on sonorant posterior probability indicate that learners did not fully achieve the approximant-like realizations characteristic of native Spanish speakers. This outcome suggested that while learners were able to approximate fricative-like lenition to some extent, the more subtle articulatory adjustments required for producing approximants remained elusive. The finding aligned with previous studies, such as those by [15], which highlighted the difficulty that L2 learners face in acquiring gradient phonological features that demand fine motor control. This difficulty may be linked to the findings of [3,7], which showed that while neuroplastic changes can support the acquisition of new phonetic contrasts, these changes may be less effective when the required articulatory adjustments are subtle and demand precise motor control.

A lack of focus on approximant-like lenition in classroom instruction might contribute to the difficulty learners experience in mastering these forms during their study-abroad program. Traditional Spanish instruction often focuses on fricative-like lenited forms, but may not sufficiently address the subtleties of phonetic gradience, particularly the approximant-like variants. Without a strong foundation in these subtleties before immersion, learners may find it challenging to acquire the necessary motor skills and perceptual awareness needed to produce these sounds accurately. This observation is supported by [21], who found that even with immersion, learners who had not received prior explicit phonetic instruction struggled to achieve native-like pronunciation in similar contexts.

The implications of these findings extend to the design and implementation of study-abroad programs. While these programs offer valuable opportunities for immersive learning and can significantly enhance certain aspects of L2 phonological acquisition, they may need to be supplemented with additional support to ensure long-term retention of these gains. This could include post-study-abroad interventions, such as continued practice with native speakers, targeted phonetic training, or the use of technology to provide ongoing exposure and feedback. Such measures could help learners maintain the progress made abroad and further develop their phonological skills to reach native-like levels.

Furthermore, the study highlighted the utility of the Phonet model in assessing L2 phonological acquisition. The model’s ability to capture gradient changes in phonological features, such as continuant and sonorant, provides a more detailed and quantitative understanding of how learners’ productions evolve over time. This approach allows researchers to move beyond traditional categorical analyses, offering a more nuanced perspective on the acquisition of phonological processes like lenition. The use of deep learning models like Phonet could be extended to other areas of L2 phonology, providing valuable insights into the mechanisms of language learning and the factors that influence successful acquisition.

In conclusion, this study demonstrated that study-abroad programs can lead to significant improvements in the acquisition of L2 phonological processes, such as lenition in Spanish voiced stops. However, the retention of these gains following study abroad is not guaranteed, and learners may revert to L1 phonological patterns in the absence of continued exposure and practice. These findings suggested that while study-abroad programs are a valuable tool for L2 acquisition, they should be complemented with ongoing support to ensure long-term success.

## 7. Limitations and Future Research

While this study offers valuable insights into the effects of a study-abroad program on the acquisition of lenition in Spanish voiced stops by native English speakers, several limitations should be noted.

First, there was no formal assessment of participants’ Spanish proficiency prior to the study-abroad program. Although all participants were in the third year of a Spanish degree program and had had substantial exposure to the language, the absence of standardized proficiency scores limits the precision with which we can gauge the initial language skills of the participants. Future research should incorporate formal assessments of proficiency to better understand how initial language abilities interact with study-abroad experiences.

Additionally, while this study considered linguistic factors, extra-linguistic variables, such as motivation, were not assessed. Previous research has shown that learners’ motivation to achieve native-like pronunciation plays a crucial role in phonological acquisition (e.g., [45,46,47]). The absence of motivational data in this study could have introduced confounding variables, as some learners may have shown greater improvement in lenition due to a stronger desire to master native-like pronunciation, while others may have lacked the same motivation. Incorporating motivational factors into future studies would provide a more comprehensive understanding of individual differences in phonological outcomes.

Another limitation is the lack of detailed information about the participants’ interaction with local speakers during the study-abroad period. While the study recorded the types of placements (university study, English teaching assistantships, and internships), it did not account for the depth or quality of interaction with native Spanish speakers, which could have significantly impacted their acquisition of lenition. Future research should examine the role of social networks and interaction patterns in phonological acquisition during study-abroad experiences.

Furthermore, the decline in lenition observed after participants returned home raises questions about the long-term retention of phonological gains. While the study shows that learners improved during immersion, the lack of sustained exposure to Spanish after returning to an English-dominant environment likely contributed to the reversion of stop-like productions. Future research should investigate the effects of post-study-abroad interventions, such as continued practice with native speakers or the use of online platforms for maintaining language exposure, to promote the retention of L2 phonological patterns.

Finally, this study utilized the Phonet model to capture gradient phonological changes, providing valuable insights into the acquisition of stop lenition. However, further validation of the model is necessary for other types of lenition. Future research should explore the application of deep learning models like Phonet to other L2 phonological processes to better assess their utility in understanding the complexities of language acquisition over time.

In conclusion, while this study contributes to our understanding of L2 phonological acquisition in a study-abroad context, addressing these limitations in future research will be essential for a more comprehensive understanding of the factors that influence successful learning and retention.

## Figures and Tables

**Table 1:** Counts of target phoneme by word position.

Phoneme	Word-Initial	Word-Medial
/p/	2617	394
/b/	1095	3644
/t/	1002	1312
/d/	2277	2128
/k/	2195	865
/ɡ/	363	155

## Data Availability

Data available upon request to the authors. The data are not publicly available due to the sensitive information contained therein.
