# Correction: Wayland et al. Lenition in L2 Spanish: The Impact of Study Abroad on Phonological Acquisition. *Brain Sci.* 2024, *14*, 946

**DOI:** 10.3390/brainsci15080817

**Published:** 2025-07-30

**Authors:** Ratree Wayland, Rachel Meyer, Sophia Vellozzi, Kevin Tang

**Affiliations:** 1 Department of Linguistics, University of Florida, Gainesville, FL 32611, USA; rmeyer2@ufl.edu; 2 Department of Computer & Information Science & Engineering, University of Florida, Gainesville, FL 32611, USA; s.vellozzi@ufl.edu; 3 Department of English Language and Linguistics, Institute of English and American Studies, Faculty of Arts and Humanities, Heinrich Heine University Düsseldorf, 40225 Düsseldorf, Germany; kevin.tang@hhu.de

## Error in Table

In the original publication [1], there was a mistake in Table 1 as published. State the mistake which was made (see below). The corrected Table 1 appears below.

## Text Correction

There was an error in the original publication [1]. Following publication, we discovered that our posterior probability analysis had inadvertently used a newly trained model on the test dataset, rather than applying the intended pre-trained Phonet model. Additionally, we revised our forced-alignment pipeline to improve token inclusion by handling pause intervals more accurately and by using the official Spanish Montreal Forced Aligner (MFA) model. As a result, the corrected analysis is based on a larger and more representative token set, as reflected in the updated Table 1 and Results section.

These changes led to revisions in several statistical outcomes, including the emergence of a significant effect of stress, reversals in some place and vowel context effects, and a shift in the comparison between native speakers and delayed post-test participants for continuant probability—now marginally significant (*p* = 0.055). However, the overall conclusions of the study remain unchanged.

A correction has been made to Sections 5.1, 5.2 and 6.

**5.**  **Results**
*5.1.*  *Continuant Posterior Probability*

As expected, there were effects of voicing (Figure 1), stress, and word position (Figure 2). Voiced stops had a higher continuant posterior probability than voiceless stops (*b* = −0.125, SE = 0.007, t = −18.155, *p* < 0.001), word-medial stops likewise had a higher continuant posterior probability than word-initial stops (*b* = 0.078, SE = 0.007, t = 12.004, *p* < 0.001), and stops in unstressed syllables had a higher continuant posterior probability than stops in stressed syllables (*b* = −0.033, SE = 0.006, t = −5.187, *p* < 0.001). There was also an effect of place, with dental stops having a lower continuant posterior probability than velar stops (*b* = −0.0.054, SE = 0.008, t = −6.449, *p* < 0.001). Finally, there was also an effect of the vowel adjacent to the target stop. Stops preceded by mid-vowels had a higher continuant posterior probability than stops preceded by close vowels (*b* = −0.143, SE = 0.005, t = −26.448, *p* < 0.001).

**Figure 1:**
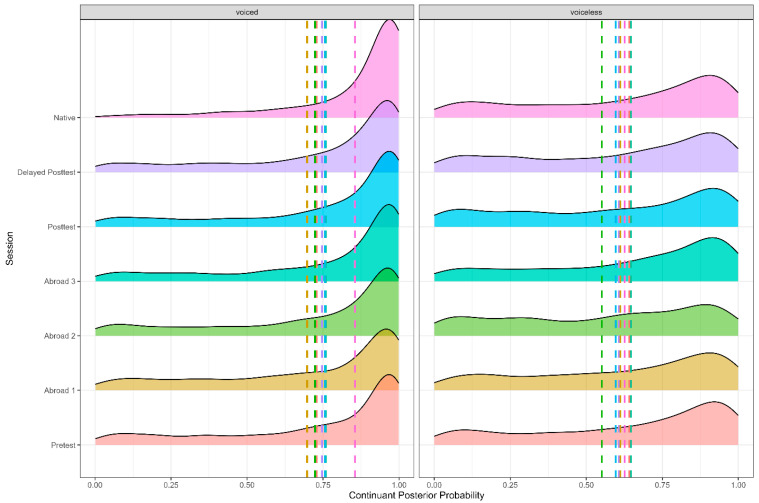
Continuant posterior probability of voiced and voiceless stops across all groups.

**Figure 2:**
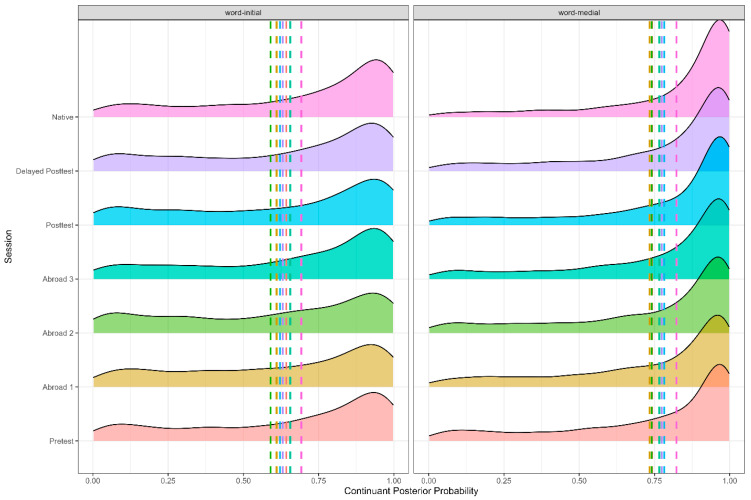
Continuant posterior probability of word-medial and word-initial stops across all groups.

Between the pre-test and the first test abroad, there was a significant decrease in posterior probability (pre-test minus first test abroad) (*b* = 0.035, SE = 0.008, t = −4.207, *p* < 0.001) (Figure 3). Posterior probability also decreased between the first and second tests abroad (*b* = 0.017, SE = 0.008, t = 2.062, *p* = 0.039), but increased between the second and third tests (*b* = −0.063, SE = 0.009, t = −7.396, *p* < 0.001) (Figure 3). There was another decrease in posterior probability between the third test and the post-test (*b* = 0.025, SE = 0.008, t = 3.150, *p* = 0.002), and no differences between post-test and delayed post-test (*b* = −0.002, SE = 0.009, t = −0.265, *p* = 0.791), and delayed post-test and native speakers, although this difference was only marginally non-significant (*b* = −0.038, SE = 0.020, t = −1.965, *p* = 0.055), with the native speaker posterior probability higher than the delayed post-test (Figure 3). The decrease–increase trajectory could suggest that participants began with a fricative production, with the continuant posterior probability as participants tried to figure out the approximant production, as evidenced by the increased posterior probability by the third test abroad. However, participants regressed by the post-test.

**Figure 3:**
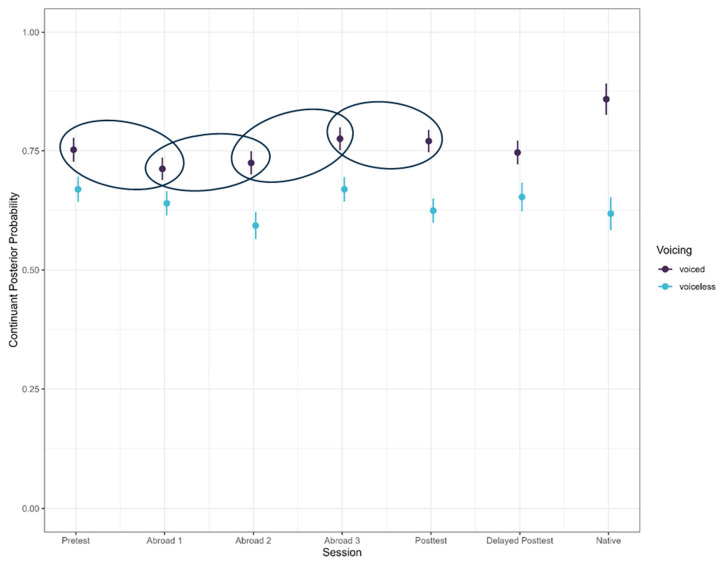
Continuant posterior probability across groups and voicing conditions. Significant comparisons are circled.

*5.2.*  *Sonorant Posterior Probability*

As expected, there was an effect of word position (Figure 4), stress, and stop voicing (Figure 5), with word-medial stops having a higher posterior probability than word-initial stops (*b* = 0.061, SE = 0.006, t = 10.519, *p* < 0.001). Voiced stops had a higher posterior probability than voiceless ones (*b* = −0.244, SE = 0.006, t = −40.175, *p* < 0.001), and stops in unstressed syllables had a higher posterior probability than stops in stressed ones (*b* = −0.012, SE = 0.006, t = −2.142, *p* = 0.032). In terms of place, there was a significant difference between bilabial and dental stops, with dental stops having a higher sonorant posterior probability (*b* = −0.012, SE = 0.006, t = −2.142, *p* = 0.032). The preceding vowel also affected the sonorant of the stop—when preceded by an open vowel, stops had a higher sonorant posterior probability than when preceded by a mid-vowel (*b* = −0.028, SE = 0.006, t = −5.012, *p* < 0.001), which in turn had a higher sonorant posterior probability than stops preceded by a close vowel (*b* = −0.183, SE = 0.006, t = −38.514, *p* < 0.001).

**Figure 4:**
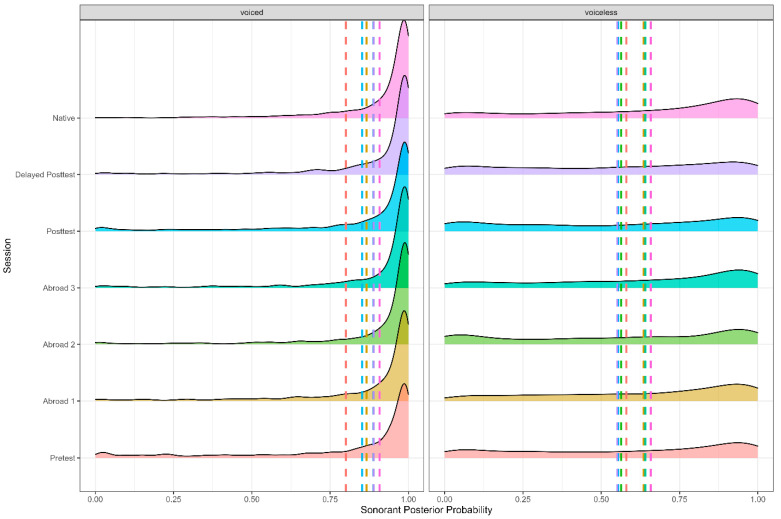
Sonorant posterior probability of voiced and voiceless stops across all groups.

**Figure 5:**
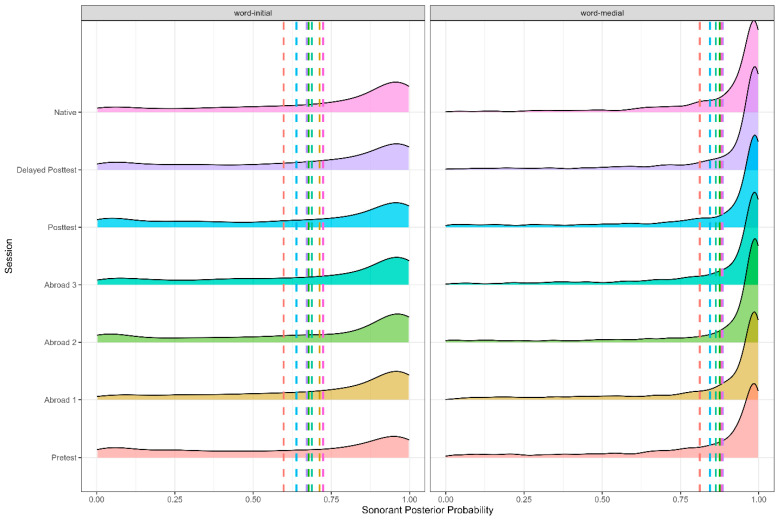
Sonorant posterior probability of word-medial and word-initial stops across all groups.

There was an increase in posterior probability between the pre-test and first test abroad (*b* = −0.044, SE = 0.007, t = −6.035, *p* < 0.001), but a decrease between the first and second tests abroad (*b* = 0.028, SE = 0.007, t = 3.788, *p* < 0.001), followed by another increase between the second and third tests abroad (*b* = −0.031, SE = 0.008, t = −4.062, *p* < 0.001), and a decrease at post-test similar to continuant (*b* = −0.030, SE = 0.007, t = 4.338, *p* < 0.001). While there was no difference between post-test and delayed post-test (*b* = 0.001, SE = 0.008, t = 0.176, *p* = 0.860), the posterior probability was significantly smaller than for native speakers (*b* = −0.053, SE = 0.015, t = −3.457, *p* = 0.001), suggesting that although L2 speakers made some improvements, they never lenited to the level of approximants (Figure 6).

**Figure 6:**
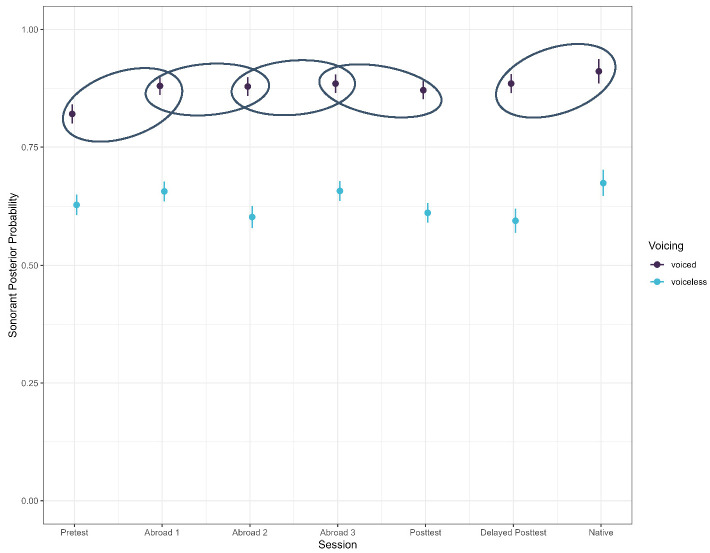
Sonorant posterior probability across groups and voicing conditions. Significant comparisons are circled.

**6.**  **Discussion and Conclusions**

This study explored the effects of a study-abroad program on the acquisition of lenition in Spanish voiced stops among native English speakers. The findings revealed that while learners made progress in adopting lenition patterns during their time abroad, their mastery remained limited, and although some gains were retained, learners did not fully maintain native-like realizations after the program. These results highlight the complex nature of L2 phonological acquisition and raise important considerations about the design and effectiveness of study-abroad programs as a means to enhance linguistic proficiency.

One of the key findings was the significant increase in continuant posterior probability during the study-abroad period, suggesting that learners became more adept at producing fricative-like lenited forms of Spanish voiced stops. This improvement indicated that immersive exposure to a native Spanish-speaking environment can facilitate the learning of subtle phonological processes, which might be difficult to acquire in a classroom setting. The naturalistic and continuous exposure to native speech, combined with the necessity to communicate effectively in the target language, likely drove these improvements. The finding aligned with previous research showing that study-abroad programs can lead to notable gains in L2 phonetic and phonological skills, particularly in areas requiring fine-grained articulatory adjustments (e.g., [21,22]).

However, the trajectory of these gains was more complex than initially believed. Rather than a monotonic increase, learners showed an early improvement, a temporary dip mid-program, and a recovery toward the later abroad sessions. Importantly, although the continuant posterior probability decreased following the return from abroad, the difference between the delayed post-test and native speaker values was marginally significant (*p* = 0.055), suggesting that learners’ productions became more similar to those of native speakers. It provides evidence that some of the gains made during study abroad may have been retained.

These findings can be further understood in the context of neuroplasticity research associated with language learning. The increase in continuant posterior probability during the study-abroad period can be viewed as a manifestation of neuroplasticity, where learners’ brains adapted to the new phonological demands of the Spanish language. This adaptation likely involved structural changes in brain regions critical for language processing, such as the inferior frontal gyrus (IFG) and superior parietal lobule (SPL), as highlighted by [5]. While there was a decline in continuant values after the study-abroad period, the marginally significant difference from native speakers at the delayed post-test suggests that some neuroplastic changes may have been retained, even after learners returned to an L1-dominant environment.

In [5], a discussion on the role of white matter integrity in managing dual language systems offers a possible explanation for the variability in lenition retention observed among participants. Learners’ ability to switch between L1 and L2 phonological patterns may depend on the efficiency of their white matter tracts, particularly those connecting language-related regions. If these tracts were less developed or less efficient, this could contribute to the decline in lenition retention after the study-abroad experience, as the cognitive control mechanisms required to sustain these gains may not have been fully established.

The Attention Model of [42] provides additional insights, emphasizing the importance of linguistic awareness in successful L2 phonological learning. The initial gains observed during the study-abroad program suggest that learners were beginning to develop this awareness. However, the subsequent decline in performance indicates that this awareness may not have been sufficiently reinforced, or that learners reverted to L1 phonological patterns once they were no longer immersed in the L2 environment. This underscores the need for targeted post-study-abroad interventions that focus on reinforcing L2 phonological patterns and enhancing learners’ ability to maintain these patterns over time.

Additionally, the role of social interaction and motivation in language learning among older adults, as discussed by [43], may also be relevant in the context of study-abroad programs. While there was a decline in performance after returning from the immersive environment, the marginally significant difference between learners and native speakers at the delayed post-test suggests that some aspects of this phonological awareness may have been retained. This underscores the need for targeted post-study-abroad interventions that not only reinforce L2 phonological patterns but also support the continued development and stabilization of learners’ phonological awareness over time.

The results regarding the effects of the place of articulation on lenition provided further nuance. Contrary to [44]’s findings, the results showed that dental stops had lower continuant posterior probabilities than velars, suggesting that the dentals were less likely to be realized as fricatives. This may be due to the anterior location and narrow constriction of dental stops, which limit the buildup and channeling of airflow required for sustained turbulent noise. In contrast, velars benefit from a longer front cavity and greater oral pressure, both of which facilitate the aerodynamic conditions necessary for frication when the constriction is partially relaxed. Dentals also showed higher sonorant posterior probabilities than bilabials, indicating a greater likelihood of approximant-like realizations. This pattern may reflect the articulatory and acoustic characteristics of bilabials: the lack of a resonant cavity in front of the constriction and strong acoustic damping at the lips reduce the potential for clear formant structure, making bilabial approximants less acoustically distinct. Together, these findings highlight how different places of articulation may favor different pathways along the lenition continuum.

In contrast to the results on continuant posterior probabilities, the results on sonorant posterior probability indicate that learners did not fully achieve the approximant-like realizations characteristic of native Spanish speakers. This outcome suggested that while learners were able to approximate fricative-like lenition to some extent, the more subtle articulatory adjustments required for producing approximants remained elusive. The finding aligned with previous studies, such as that by [15], which highlighted the difficulty L2 learners face in acquiring gradient phonological features that demand fine motor control. This difficulty may be linked to the findings of [3,7], which showed that while neuroplastic changes can support the acquisition of new phonetic contrasts, these changes may be less effective when the required articulatory adjustments are subtle and demand precise motor control.

A lack of focus on approximant-like lenition in classroom instruction might contribute to the difficulty learners experience in mastering these forms during their study-abroad program. Traditional Spanish instruction often focuses on fricative-like lenited forms, but may not sufficiently address the subtleties of phonetic gradience, particularly the approximant-like variants. Without a strong foundation in these subtleties before immersion, learners may find it challenging to acquire the necessary motor skills and perceptual awareness needed to produce these sounds accurately. This observation is supported by [21], who found that even with immersion, learners who had not received prior explicit phonetic instruction struggled to achieve native-like pronunciation in similar contexts.

Additionally, the analysis revealed that unstressed syllables elicited significantly higher continuant and sonorant posterior probabilities than stressed syllables, a pattern not observed in our original report. This is consistent with broader findings in phonetics showing that lenition is more likely in prosodically weak positions. Likewise, the revised vowel context effect showed that open vowels were associated with greater sonorant posterior probabilities than mid- or close vowels. Together, these updated findings highlight the importance of considering prosodic and segmental context in the acquisition of lenition patterns.

The implications of these findings extend to the design and implementation of study-abroad programs. While these programs offer valuable opportunities for immersive learning and can significantly enhance certain aspects of L2 phonological acquisition, they may need to be supplemented with additional support to ensure long-term retention of these gains. This could include post-study-abroad interventions, such as continued practice with native speakers, targeted phonetic training, or the use of technology to provide ongoing exposure and feedback. Such measures could help learners maintain the progress made abroad and further develop their phonological skills to reach native-like levels.

Furthermore, the study highlighted the utility of the Phonet model in assessing L2 phonological acquisition. The model’s ability to capture gradient changes in phonological features, such as continuant and sonorant, provides a more detailed and quantitative understanding of how learners’ productions evolve over time. This approach allows researchers to move beyond traditional categorical analyses, offering a more nuanced perspective on the acquisition of phonological processes like lenition. The use of deep learning models like Phonet could be extended to other areas of L2 phonology, providing valuable insights into the mechanisms of language learning and the factors that influence successful acquisition.

In conclusion, this study demonstrated that study-abroad programs can lead to significant improvements in the acquisition of L2 phonological processes, such as lenition in Spanish voiced stops. While earlier findings suggested that learners reverted to more stop-like productions post-study-abroad, the corrected analysis indicates that some of these gains may have been more durable than originally thought, as evidenced by the near-significant difference between delayed post-test and native speaker values. Nonetheless, learners did not fully achieve native-like approximant realizations, and retention remained incomplete without continued exposure. These findings suggest that while study-abroad programs are a valuable tool for L2 acquisition, they should be complemented with ongoing support to ensure long-term success.

The authors state that the scientific conclusions are unaffected. This correction was approved by the Academic Editor. The original publication has also been updated.

## Figures and Tables

**Table 1:** Counts of target phoneme by word position.

Phoneme	Word-Initial	Word-Medial
/p/	3530	469
/b/	1543	2522
/t/	1174	1253
/d/	2661	2522
/k/	3799	744
/ɡ/	1438	1506
